# Impact of the COVID‐19 pandemic on international cutaneous squamous cell carcinoma incidence: A systematic review and meta‐analysis

**DOI:** 10.1002/ski2.405

**Published:** 2024-06-15

**Authors:** James Wall, Kieran Gadsby‐Davis, Khaylen Mistry, Nick J. Levell, Zoe C. Venables

**Affiliations:** ^1^ University of East Anglia Norwich Medical School Norwich UK; ^2^ Dermatology Norfolk and Norwich University Hospitals NHS Foundation Trust Norwich UK

## Abstract

**Background:**

Non‐melanoma skin cancer (NMSC) is the most common cancer globally in white ethinicity populations, and cutaneous squamous cell carcinoma (cSCC) is the second most common subtype. The COVID‐19 pandemic severely impacted public and private healthcare systems. Many studies have reported reduced cancer diagnoses during the pandemic. The impact of the COVID‐19 pandemic on global cSCC and NMSC incidence is poorly reported.

**Objectives:**

The aim was to conduct a systematic review and meta‐analysis to assess the impact of the COVID‐19 pandemic on global cSCC and NMSC incidence rates, compared with 2019 incidence rates. Two primary outcome measures were used: crude incidence rate ratios (CIRR) and age‐standardised incidence rate ratios (ASIRR).

**Methods:**

A structured search was undertaken on 23 March 2023 using grey literature and four electronic databases: MEDLINE, CINAHL, EMBASE and Web of Science. Studies published before January 2020 were excluded. A quality assessment was undertaken using A. Lomas quality assessment tool. CIRR outcomes were synthesised in a meta‐analysis, while ASIRR outcomes were narratively synthesised.

**Results:**

Fourteen cancer registries were included, capturing data from 13 countries across Europe. Variation was observed in NMSC and cSCC incidence across the cancer registries. Pooled cSCC crude incidence rates in 2020 were equal to crude incidence rates in 2019 (cSCC‐CIRR 1.00 (95% confidence interval (CI) 0.94–1.06). In 2021, the pooled result indicated a non‐significant 8% increase in cSCC crude incidence rates, compared with 2019 (cSCC‐CIRR 1.08 (95% CI 0.98–1.19). Significant reductions were reported in NMSC incidence across all meta‐analyses in 2020 and 2021 compared with 2019. Heterogeneity was observed across most pooled estimates (*I* ^2^>75%).

**Conclusion:**

There was a lack of high quality data on cSCC incidence rates recorded during the pandemic outside of Europe. The COVID‐19 pandemic resulted in no significant changes in cSCC incidence across Europe. By contrast, NMSC incidence fell across Europe following the pandemic. Significant reductions in pooled NMSC incidence rates may reflect a delay in basal cell carcinoma presentation, diagnosis and treatment. Although annual incidence rates for cSCC were not affected by the pandemic, delays in treatment may still have occurred, which may result in poorer outcomes yet to be fully understood.

**What is already known about this topic?**

- Incidence rates of cutaneous squamous cell carcinoma (cSCC) and Non‐melanoma skin cancer (NMSC) are high and increasing in most countries worldwide.
- NMSC incidence data are often poorly recorded worldwide.
- The COVID‐19 pandemic impacted healthcare systems globally resulting in reductions in recorded incidence of some cancers.
- The impact of the COVID‐19 pandemic on NMSC incidence rates is not well reported.

**What does this study add?**

- cSCC incidence rates showed no significant change in 2020 and 2021 compared with 2019 in 13 countries across Europe.
- NMSC incidence rates were lower in 2020 and 2021 compared to 2019 in 13 countries across Europe. This suggests there may have been delayed diagnosis of non melanoma skin cancers other than squamous cell carcinoma in these countries.

## 1 BACKGROUND

Non‐melanoma skin cancer (NMSC) predominantly comprises basal cell carcinoma (BCC) and cutaneous squamous cell carcinoma (cSCC).^1^ BCC accounts for ∼75%, while cSCC represents ∼25%.^2^

Globally, many cancer registries have inadequate registration practices for reporting NMSC incidence, either by not reporting any NMSC data, not detailing histological subtypes, or failing to report more than one tumour per patient.^3^, ^4^, ^5^, ^6^, ^7^ Despite this, NMSC incidence was the most common cancer globally in 2017, with cSCC incidence estimated at 2.4 million in 2019.^8^, ^9^ The rising incidence of cSCC has a high economic burden.^2^, ^10^, ^11^

The COVID‐19 pandemic severely impacted public and private healthcare systems globally.^12^ To reduce COVID‐19 mortality, countries implemented lockdowns, promoted stay at home campaigns, and advised vulnerable populations such as the elderly or immunocompromised to shield themselves.^13^, ^14^ Hospital staff were redeployed to frontline services and cancer services were restricted.^15^ This was associated with a sharp decline in all cancer diagnoses in April 2020, with cancer diagnoses recovering to pre‐pandemic levels by June–October 2020 according to one global systematic review.^16^ This aligns with cSCC incidence studies assessing the earlier months of the pandemic in 2020.^17^, ^18^, ^19^, ^20^, ^21^, ^22^, ^23^ Factors such as the public fearing to attend medical care and restrictions in accessing healthcare for non‐urgent appointments may have influenced this decline.^18^, ^19^ Moreover, organisations such as the American College of Mohs Surgery advised delaying lower risk surgeries for up to 3 months to balance COVID‐19 risks with cancer outcomes.^13^ Previous reports indicated substantial variation in cSCC incidence returning to pre‐pandemic levels, possibly due to differences in re‐introducing elective surgery and the utilisation of telemedicine and teledermatology between countries.^24^, ^25^, ^26^, ^27^, ^28^, ^29^, ^30^

The World Health Organisation (WHO) NMSC incidence report in 2020 extrapolated pre‐pandemic data, therefore current international NMSC incidence rates are unknown.^31^ This systematic review assessed whether cSCC and NMSC incidence rates in 2020 and 2021 changed due to the impact of the COVID‐19 pandemic.

## 2 METHODS

### 2.1 Registration

The protocol was registered on PROSPERO (ID: CRD42022376497). The review was reported according to the Meta‐analyses Of Observational Studies in Epidemiology statement.

### 2.2 Eligibility criteria

Studies were included if they reported cSCC and/or NMSC incidence rates, or provided adequate data to calculate incidence rates, both before and after 1 January 2020, which aligns with WHO's recognition of SARS‐CoV‐2 cases.^12^ No restrictions were applied on study design. Studies on specific populations such as genetic syndromes or renal transplant patients were excluded. Studies only providing estimated incidence rates after 1 January 2020 were excluded. Studies reporting data in the same population occurring at the same time point were assessed, with only one study being included.

### 2.3 Literature search

A comprehensive search was performed on 23 March 2023. Four electronic databases were searched systematically using date restrictions between 2020 and March 2023 (MEDLINE, CINAHL, EMBASE, Web of science). Date restrictions were applied to meet the eligibility criteria.^32^ Search strategy focused on the main concepts: cSCC, NMSC, COVID‐19, and epidemiology.^33^ Grey literature was searched on Google Scholar and Google. Thirty‐three countries, classified with high‐quality epidemiological data by the Global Burden of Disease (GBD) study, were screened using Google for cancer registries or government reports.^34^ No language restrictions were imposed on search strategy results and translations were obtained. See Table S1 for full search strategy.

After removing duplicates, two reviewers (JW, KGD) independently screened the titles and abstracts of all studies to assess eligbility. Both reviewers independently conducted full‐text reading on the studies considered potentially eligible. Uncertainties were resolved by a third reviewer (ZV/NJL).

### 2.4 Data extraction

Full data extraction was conducted independently by two reviewers (JW, KGD). A data extraction template was created for the review and was used to extract: author, year, country, setting, study design, sex, diagnosis, method of confirmation, and crude and/or age‐standardised incidence rates. Corresponding authors were emailed for missing information and non‐English studies were translated. Discrepancies between reviewers were resolved via a third reviewer (ZV/NJL).

### 2.5 Quality assessment

After a pilot assessment, a quality assessment was conducted independently by two reviewers (JW, KGD) using a tool previously by published by Lomas et al.^2^ The tool consisted of 10 questions, each equally weighted. Discrepancies were resolved with a third reviewer (ZV/NJL).

### 2.6 Synthesis of results

Two primary outcomes compared cSCC and/or NMSC incidence rates during the 2020–21 COVID‐19 pandemic with 2019 incidence rates to determine incidence rate ratios. Incidence rates are expressed per 100 000 person‐years. Separate analyses for cSCC and NMSC data were performed, and studies reporting both were included in both analyses. Studies reporting the International Classification of Diseases, 10th Edition (ICD‐10) code ‘C44 excluding BCC’ were included in the cSCC analyses.^35^

### 2.7 Meta‐analysis

Meta‐analyses were performed on crude incidence rate ratios (CIRR). The inverse variance statistical method was applied, and due to the differential impact of the pandemic between populations, a random effects model was used.^36^ Four forest plots were generated using Review Manager version 5.4.1.^37^ Statistical heterogeneity was evaluated using the Higgins *I* ^2^ statistic, with considerable heterogeneity indicated if *I* ^2^>75%.^36^ CIRRs were calculated using the formula:

- $\frac{\left(\left(\text{cSCC}\;\text{diagnoses}\;\text{during}\;\text{COVID}-19\times 100,000\right)÷\text{population}\;\text{at}\;\text{risk}\right)}{\left(\left(\text{cSCC}\;\text{diagnoses}\;\text{pre}-\text{COVID}-19\times 100,000\right)÷\text{population}\;\text{at}\;\text{risk}\right)}=\frac{\left(\text{cSCC}\;\text{crude}\;\text{incidence}\;\text{rate}\;\text{during}\;\text{COVID}-19\right)}{\left(\text{cSCC}\;\text{crude}\;\text{incidence}\;\text{rate}\;\text{in}\;2019\right)}$

The natural logarithm of the CIRR and standard error were calculated and entered into Review Manager.^38^

### 2.8 Narrative synthesis

Age‐standardised incidence rate ratio (ASIRR) were narratively synthesised as all studies could not be compared to the same standardised population structure due to inadequate age‐group specific incidence data between included studies. ASIRRs were calculated using the formula:

- $\frac{\left(cSCC\;age-standardised\;incidence\;rate\;during\;COVID-19\right)}{\left(cSCC\;age-standardised\;incidence\;rate\;in\;2019\right)}$

Statistical differences between sex were determined using Poisson method.

## 3 RESULTS

### 3.1 Study selection

**FIGURE 1:**
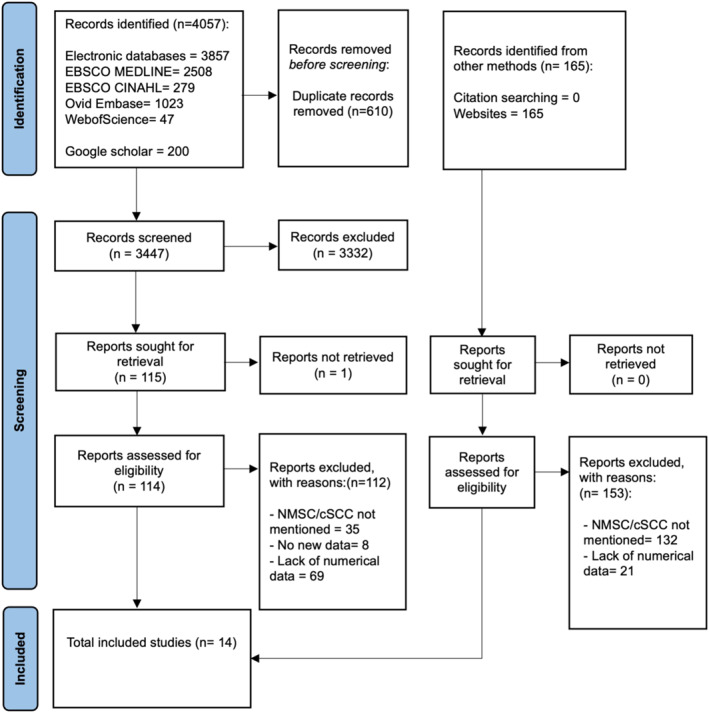
PRISMA flow diagram of literature search and study selection process. A search was conducted on 23rd March 2023, including grey literature and four bibliographic databases. Three thousand, four hundred forty‐seven studies were initially screened with 14 studies meeting the eligibility criteria and included for analysis.

The study selection process is outlined using a PRISMA flow diagram (Figure 1).^39^ A total of 14 studies were included in the review.

### 3.2 Study characteristics

Table 1 describes the characteristics of studies included. The review encompassed 14 cancer registries across Europe. All studies reported CIRRs, of which 11 provided ASIRRs. The COVID‐19 pandemic group included study data recorded between January 2020 and December 2022. The median study duration in the pandemic group was 365 days (IQR = 365 days). cSCC data were reported in six studies, NMSC data in seven studies, and four studies used the ICD‐10 C44 classification (NMSC) excluding BCC. Sex‐specific data was available in 12 studies.

**TABLE 1:** Summary of characteristics of all included studies.

Author, year	Population	Study design	Sex	Diagnosis	Outcomes reported	Standard population
Belgium, 2023	Belgium	Cancer registry	B, M, F	cSCC and NMSC	CIRR, ASIRR	European 2013
Biscgelia, 2022	Reggio Emilia, Italy	Cancer registry	B	NMSC	CIRR	No standardisation used
Denmark, 2023	Denmark	Cancer registry	B	ICD‐10 C44 exc. BCC	CIRR	No standardisation used
NHS digital, 2022	England	Cancer registry	B, M, F	NMSC	CIRR, ASIRR	European 2013
NCR, 2022	Netherlands	Cancer registry	B, M, F	cSCC	CIRR, ASIRR	European 2013
NORDCAN, 2022	Iceland	Cancer registry	B, M, F	ICD‐10 C44 exc. BCC	CIRR, ASIRR	Nordic 2000
NORDCAN, 2022	Sweden	Cancer registry	B, M, F	ICD‐10 C44 exc. BCC	CIRR, ASIRR	Nordic 2000
Northern Ireland, 2020	Northern Ireland	Cancer registry	B, M, F	NMSC	CIRR, ASIRR	European 2013
Norway, 2021	Norway	Cancer registry	B, M, F	ICD‐10 C44 exc. BCC	CIRR, ASIRR	European 1976
Pitkäniemi, 2020	Finland	Cancer registry	B, M, F	cSCC	CIRR, ASIRR	Finland 2014
Ribes, 2022	Catalonia, Spain	Cancer registry	B, M, F	cSCC	CIRR	No standardisation used
Saarland, 2022	Saarland, Germany	Cancer registry	B, M, F	NMSC	CIRR, ASIRR	European 1976
Schleswig‐Holstein, 2022	Schleswig‐Holstein, Germany	Cancer registry	B, M, F	cSCC and NMSC	CIRR, ASIRR	European 1976
Scotland, 2021	Scotland	Cancer registry	B, M, F	cSCC and NMSC	CIRR, ASIRR	European 2013

Abbreviations: ASIRR, age‐standardised incidence rate ratios; B, both male and female data recorded together; BCC, basal cell carcinoma; CIRR, crude incidence rate ratio; cSCC, cutaneous squamous cell carcinoma; F, female only; M, male only; NMSC, non‐melanoma skin cancer.

### 3.3 Quality assessment

The methodological quality of the studies varied with a range of scores between two and eight. Twelve (86%) were deemed of high quality (score ≥6).^2^ However, no study met all the criteria, directly sampled the population, as opposed to using cancer registries, and no study provided information about ethnicity or skin type. All studies included histological verification, as European cancer registries consider the ‘most valid basis of diagnosis’, such as histology, in counting a cancer case.^7^ Table S2 reports quality assessment results.

### 3.4 Crude incidence rate ratios (CIRR)

**FIGURE 2:**
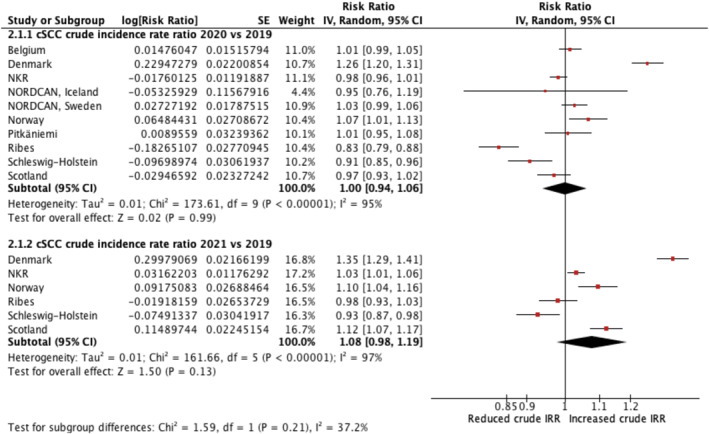
Forest plot reporting cSCC crude incidence rate ratios: comparing data recorded during 2020 and 2021 against cSCC crude incidence rates in 2019. cSCC, cutaneous squamous cell carcinoma.

**FIGURE 3:**
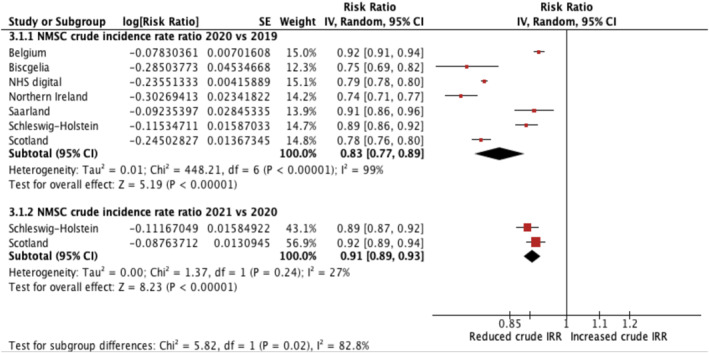
Forest plot reporting NMSC crude incidence rate ratios comparing 2020 and 2021 crude incidence rates against NMSC crude incidence rates in 2019. NMSC, non‐melanoma skin cancer.

A summary table of results for CIRRs is provided in Table S3. The meta‐analysis results for cSCC‐CIRR and NMSC‐CIRR are displayed in forest plots in Figures 2 and 3, respectively. All meta‐analyses, except one, indicated considerable heterogeneity (*I* ^2^>75%) in each yearly CIRR group.

### 3.5 cSCC‐CIRR

Ten studies compared cSCC incidence in 2020 against 2019. This covered a total population area of approximately 72 million, encompassing eight national studies and two regional studies. Compared with 2019, Catalonia, Spain, reported the greatest decrease cSCC‐CIRR of 0.83 (confidence interval (CI) 0.79–0.88), whereas Denmark had the greatest increase in cSCC crude incidence rates (cSCC‐CIRR 1.26, CI 1.20–1.31). The pooled result for cSCC‐CIRR was 1.00 (CI 0.94–1.06), suggesting a non‐significant change between pooled cSCC crude incidence rates in 2020 compared to 2019.

Six studies compared cSCC incidence in 2021 against 2019. This covered a total sample size of approximately 45 million, encompassing four national and two regional studies. Schleswig‐Holstein, Germany, was the only study with a significant decrease in cSCC‐CIRR of 0.93 (CI 0.87–0.98). By contrast, Denmark reported 35% higher cSCC crude incidence rates in 2021, compared with 2019 (cSCC‐CIRR 1.35, CI 1.29–1.41). The pooled result of cSCC‐CIRR was 1.08 (CI 0.98–0.1.19), representing a non‐significant 8% increase in cSCC crude incidence rates in 2021 compared with 2019. Scotland and Catalonia, Spain, reported significantly higher cSCC‐CIRR in 2021 compared with 2020.

### 3.6 NMSC‐CIRR

Seven studies compared NMSC incidence in 2020 against 2019 which included four national, two regional, and one local study. A total population area of approximately 80 million was captured. Northern Ireland reported the greatest decrease in NMSC‐CIRR (0.74, CI 0.71–0.77), whereas Belgium had a smaller but still significant decreases in NMSC‐CIRR (0.92, CI 0.91–0.94). The pooled NMSC‐CIRR was 0.83 (CI 0.77–0.89). This suggested a significant reduction in NSMC crude incidence rates in 2020 compared to 2019.

Two studies compared NMSC incidence in 2021 against 2019 which covered a total population size of approximately 8 million. Schleswig‐Holstein in Germany reported a significant decrease in NMSC‐CIRR of 0.89 (CI 0.87–0.92). Similarly, Scotland, reported reductions of 0.92 (CI 0.89–0.94). The pooled NMSC‐CIRR was 0.91 (CI 0.89–0.93), suggesting a significant decrease in NMSC crude incidence rates in 2021 compared to 2019. *I* ^2^ = 27% suggested minimal heterogeneity.

### 3.7 Age‐standardised incidence rate ratios (ASIRR)

A summary table of results for ASIRR is provided in Table S4. Eleven cancer registries in Europe were included. All studies reported male and female data separately.

### 3.8 cSCC‐ASIRR

**FIGURE 4:**
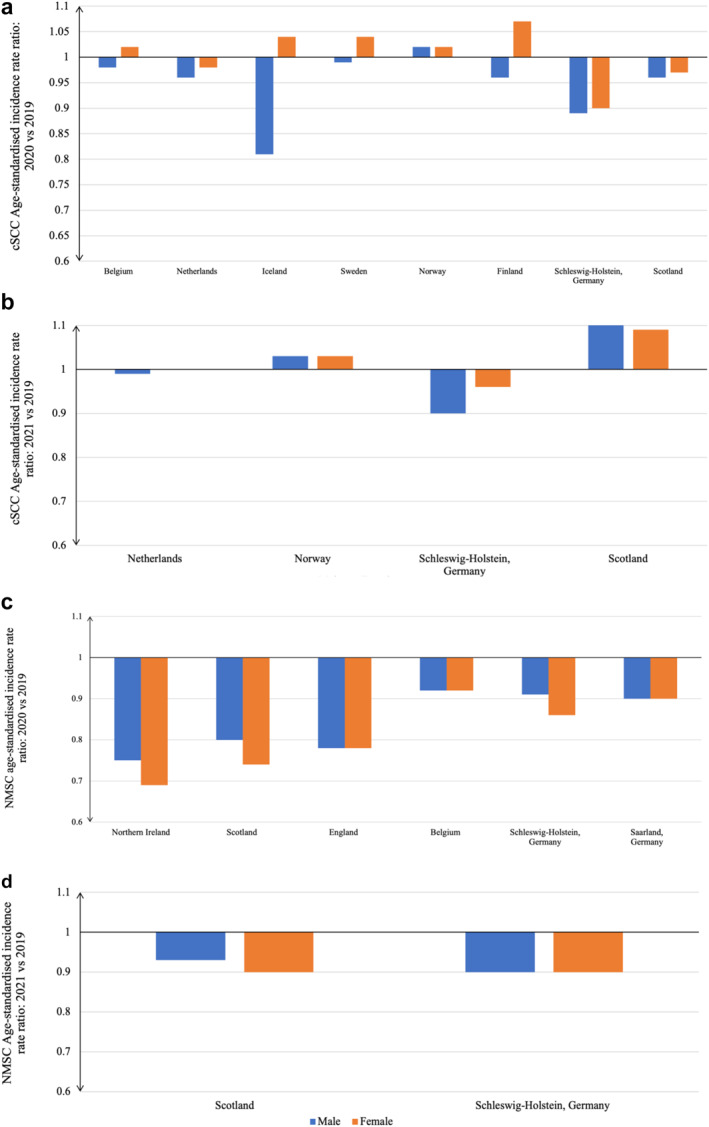
Summary of findings for cSCC and NMSC age‐standardised incidence rate ratios. (a) Comparing cSCC age‐standardised incidence rates in 2020 against 2019 in eight studies; (b) Comparing cSCC age‐standardised incidence rates in 2021 against 2019 in four studies; (c) Comparing NMSC age‐standardised incidence rates in 2020 against 2019 in six studies; (d) Comparing NMSC age‐standardised rates in 2021 against 2019 in two studies. cSCC, cutaneous squamous cell carcinoma; NMSC, non‐melanoma skin cancer.

Eight studies compared cSCC incidence in 2020 against 2019 which included seven national studies and one regional study. Various standard populations were reported: ESP 2013 (*n* = 3), ESP 1976 (*n* = 2), NORDCAN 2000 (*n* = 2), and Finland 2014 (*n* = 1). Figure 4a indicates ASIRR scores ranged between 0.81 and 1.02 in males and between 0.90 and 1.07 among females, however this difference was non‐significant (*p* = 0.08). Only four studies reported cSCC‐ASIRR for the whole population. Belgium reported a 1% increase (ASIRR 1.01), Finland reported no change, and two studies reported reduced ASIRR (Scotland (0.97), Netherlands (0.97)).

Four studies compared cSCC incidence in 2021 against 2019 which included three national and one regional study. The standard populations reported were: ESP 2013 (*n* = 2) and ESP 1976 (*n* = 2). Figure 4b demonstrates that cSCC‐ASIRR scores ranged between 0.90 and 1.10 in males and between 0.96 and 1.09 in females. All studies reported higher cSCC‐ASIRRs in 2021 compared to cSCC‐ASIRRs in 2020, with the greatest increase observed in Scotland. Two studies reported cSCC‐ASIRR for the whole population. Netherlands reported a cSCC‐ASIRR of 1.00 while Scotland reported a cSCC‐ASIRR of 1.09.

### 3.9 NMSC‐ASIRR

Six studies compared NMSC incidence in 2020 against 2019 which included four national and two regional studies. The standard populations reported were: ESP 2013 (*n* = 4) and ESP 1976 (*n* = 2). Figure 4c demonstrates that NMSC‐ASIRR decreased comparably in both sexes. ASIRR scores ranged between 0.75 and 0.92 in males and between 0.69 and 0.92 among females. Four studies reported NMSC‐ASIRR for the whole population. The NMSC‐ASIRR scores ranged between 0.73 and 0.92. The lowest and highest NMSC‐ASIRR was reported in Northern Ireland and Belgium, respectively.

Two studies compared NMSC incidence in 2021 against 2019 (Figure 4d). Scotland and Schleswig‐Holstein. NMSC‐ASIRR scores ranged between 0.90 and 0.93 in males and between 0.86 and 0.90 among females.

## 4 DISCUSSION

This systematic review assessed 14 studies on the impact of the COVID‐19 pandemic on cSCC and NMSC incidence rates.

The pooled result for cSCC‐CIRR reported a 0% change between 2020 and 2019 crude incidence rates. By contrast most cancers, of any type, had been reported as having lower incidence rates in 2020.^40^, ^41^ The presentation of cSCC is rapid in onset and often painful therefore diagnosing most cases of cSCC either face‐to‐face or via telemedicine is not challenging for experienced clinicians. Delayed presentation during the pandemic may have been less likely for cSCC than for other cancers with less obvious visual or sensory impact resulting in a lower threshold for seeking urgent medical attention. However, before the pandemic, cSCC incidence increased consistently year on year in most populations globally, and predictions indicated continued upward trends.^2^, ^10^, ^42^ Given this trend, no change between cSCC incidence in 2019 and 2020 may reflect a reduction in cSCC presentation by patients. Furthermore, our findings suggested lower cSCC‐ASIRR in males than females in 2020, compared to 2019 although this was non‐significant. This requires further research but may be due to differences in health seeking behaviours between sexes during the pandemic.^43^ Delays in cSCC could lead to fatal outcomes.^44^

Pooled estimates indicated significant reductions in NMSC incidence rates in 2020 and 2021, compared with 2019. However pooled estimates for changes in cSCC incidence rates lacked significance. Approximately 75% of NMSC cases constitute BCC, suggesting the reductions mainly involved BCC.^45^ The pandemic may have impacted BCC and cSCC incidence differently due to differences in care pathways.^46^, ^47^ BCCs may have been managed within more routine care pathways which faced larger delays.^48^, ^49^ By contrast, cSCC may have prioritised on emergency and cancer pathways during the pandemic.^48^, ^49^ Furthermore, BCC's slower development and less symptomatic growth than cSCC could have led to fewer individuals seeking medical attention.^47^ Lastly, BCC is often diagnosed incidentally during whole body skin examinations, which were less frequent during the pandemic.^50^, ^51^

Higher cSCC and NMSC incidence rate ratios were observed in 2021 compared with 2020, suggesting a trajectory towards pre‐pandemic levels of healthcare service provision. This trend is supported by WHO data which indicated reduced excess mortality in 2021, a measure used to assess the impact of the COVID‐19 pandemic.^52^, ^53^ Teledermatology is likely to have mitigated service delays for some diseases given its high satisfaction rates and government‐funding in some countries.^28^, ^29^, ^54^, ^55^, ^56^ cSCC may be more common in frail individuals who had high COVID‐19 mortality which may have partially influenced the impact of the pandemic on cSCC incidence rates.^47^ Despite this, cSCC incidence rates in 2021 were not significantly higher than 2019, although this could be due partially to a lack of reported data.

Several studies assessing the earlier months of 2020 have reported an increased proportion of higher‐risk cSCCs, compared to 2019 data.^17^, ^23^, ^57^ Various factors were associated with a larger tumour diameter in these studies. This included older age, residency in nursing homes, limited exposure to skin cancer campaigns during the lockdowns and and delays in patients seeking initial GP consultations.^17^, ^57^ Conversely, studies assessing the longer term impact of the pandemic on cSCC tumour characteristics have yielded heterogenous results. A Netherlands study analysing national data revealed no significant changes in cSCC tumour stage distribution, whereas several local studies in Europe have reported an increased number of thicker cSCC tumours, therefore potentially impacting patient outcomes.^25^, ^58^, ^59^

### 4.1 Strengths and limitations

CIRRs do not adjust for differences in age structure between studies. However, comparing closely matched dates in the meta‐analyses minimises potential changes in age structures. Additionally, higher mortality rates observed among individuals over 65 during the pandemic may offset the increasing age trend in populations, mitigating potential bias introduced.^60^ ASIRRs outcomes removed the confounding effect of age on NMSC diagnosis, and scored higher in the quality assessment as most standardised data to a major population, increasing the external validity.^61^ However, fewer studies were available for analysis, leading to less conclusive and generalisable results.

Considerable statistical heterogeneity (*I* ^2^>75%) was observed in most pooled estimates suggesting variability in the effect estimates rather than random error alone.^36^ Clinical heterogeneity arised from the inclusion of various countries and settings, each with different population densities and varying lockdown periods.^62^ Methodological diversity is present due to variation between countries in the quality of recording of skin cancer data. Cancer registries may underestimate incidence by not recognising multiple cSCCs in one individual.^7^ Furthermore, challenges in data collection during the early stages of the pandemic may further contribute to information bias.^63^ Exploration of heterogeneity was restricted as most included limited demographic data, some studies were relatively small and some meta‐analyses included a limited number of studies.^36^

The search strategy aimed to have a global representation but all of the publications which met the eligibility criteria were from Europe. The grey literature search focused on 33 countries, selected based on the 5‐star mortality data rating from the GBD and mainly included high‐income, white ethnicity countries.^34^ This limits how representative the data may be on a global perspective not only because incidence rates vary significantly globally for cSCC but also the response to the pandemic and healthcare service provision varied. Furthermore, data collection from private healthcare to cancer registries is unclear, possibly underestimating incidence during the pandemic considering increased private healthcare utilisation was reported.^64^

Another limitation is that a publication bias assessment wasn't performed. Many cancer registries were included, which follow strict publication policies, and the limited number of cohort studies in each primary outcome prevented Egger's Test or funnel plot analysis.^65^, ^66^

The COVID‐19 pandemic led to higher mortality rates in the frail elderly population who are more susceptible to developing cSCC.^59^ As a result we might have expected cSCC incidence to fall because of this effect but observed data did not confirm this. Therefore due to potential bias the results should be interpreted with caution.

The long‐term impact of the pandemic on cSCC incidence remains unclear. Global travel restrictions may reduce ultraviolet exposure and cSCC incidence, while encouragement for outdoor exercise may increase cSCC incidence.^67^ Other vairables such as changes in sun protection behaviours and climate change may also impact cSCC incidence, complicating attribution to the pandemic alone.^68^

### 4.2 Future research

There was a lack of high quality cSCC data from countries outside Europe. Further research should compare studies using age‐standardised incidence rates with the same major population structure enabling more accurate comparisons between countries. Furthermore, future studies should assess the potential impact of delayed cSCC diagnoses on patient outcomes, aligning with James Lind Alliance priorities.^69^ This should include assessing the effectiveness of teledermatology in managing diagnostic delays, given its limitations such as accessibility.^30^

In future pandemics, public health campaigns should increase NMSC awareness and mitigate fear of accessing healthcare to prevent delays in seeking medical attention and ensure all aspects of healthcare services continue to function.^70^, ^71^, ^72^

Lastly, alignment of NMSC epidemiology with other invasive diseases is required. Governmental support is vital in improving electronic record systems and standardised data collection globally.

## 5 CONCLUSION

In conclusion, evidence indicates that the impact of the COVID‐19 pandemic on cSCC incidence varied across Europe but appeared to be reducing with time. The available data showed no difference in cSCC crude incidence rates between 2020 and 2019 and suggested a non‐significant increase of 8% in 2021 compared to 2019. Significant reductions were reported in NMSC incidence rates in 2020, suggesting a backlog in BCC cases. The generalisability of these findings is limited due to high statistical heterogeneity, a lack of age‐standardised data, and the scarcity of cSCC incidence rates recorded during the pandemic outside of Europe. Further research is needed to explore the long‐term impact of the pandemic on cSCC outcomes.

## CONFLICT OF INTEREST STATEMENT

N.J.L is a trustee of the British Association of Dermatologists (BAD).

## AUTHOR CONTRIBUTIONS

**James Wall**: Data curation (lead); formal analysis (lead); investigation (lead); methodology (lead); project administration (lead); resources (lead); writing – original draft (lead); writing – review & editing (lead). **Kieran Gadsby‐Davis**: Data curation (supporting); formal analysis (supporting); methodology (supporting); writing – review & editing (supporting). **Khaylen Mistry**: Formal analysis (supporting); investigation (supporting); methodology (supporting); supervision (equal); visualization (equal); writing – review & editing (equal). **Nick J. Levell**: conceptualization (equal); formal analysis (equal); methodology (equal); supervision (equal); validation (equal); writing – original draft (equal); writing – review & editing (equal). **Zoe C. Venables**: conceptualization (equal); data curation (equal); formal analysis (equal); methodology (equal); resources (equal); supervision (equal); validation (equal); visualization (equal); writing – original draft (equal); writing – review & editing (equal).

## ETHICS STATEMENT

Not applicable.

## PATIENT CONSENT

Not applicable.

## Supporting information

Supporting Information S1

## Data Availability

The data underlying this article will be shared on reasonable request to the corresponding author.

## References

[1] Que SKT , Zwald FO , Schmults CD . Cutaneous squamous cell carcinoma: incidence, risk factors, diagnosis, and staging. J Am Acad Dermatol. 2018;78(2):237–247. 10.1016/j.jaad.2017.08.059 29332704

[2] Lomas A , Leonardi‐Bee J , Bath‐Hextall F . A systematic review of worldwide incidence of nonmelanoma skin cancer. Br J Dermatol. 2012;166(5):1069–1080. 10.1111/j.1365-2133.2012.10830.x 22251204

[3] World Cancer Health Research Fund International . Skin cancer statistics. https://www.wcrf.org/cancer‐trends/skin‐cancer‐statistics/. Last Accessed 5th July 2023.

[4] American Cancer Society . Key statistics for basal and squamous cell skin cancers. https://www.cancer.org/cancer/types/basal‐and‐squamous‐cell‐skin‐cancer/about/key‐statistics.html#:~:text=According%20to%20one%20estimate%2C%20about,cell%20cancers%20occur%20less%20often. Last Accessed 4th April 2023.

[5] Cancer in Australia. Cancer in Australia 2021. https://www.aihw.gov.au/getmedia/0ea708eb‐dd6e‐4499‐9080‐1cc7b5990e64/aihw‐can‐144.pdf.aspx?inline=true. Last Accessed 4th July 2023.

[6] van Bodegraven B , Vernon S , Eversfield C , Board R , Craig P , Gran S , et al. ‘Get Data Out’ Skin: national cancer registry incidence and survival rates for all registered skin tumour groups for 2013–2019 in England. Br J Dermatol. 2023;188(6):777–784. 10.1093/bjd/ljad033 36814132

[7] International Agency for Research on Cancer . Standards and guidelines for cancer registration in Europe. https://publications.iarc.fr/Book‐And‐Report‐Series/Iarc‐Technical‐Publications/Standards‐And‐Guidelines‐For‐Cancer‐Registration‐In‐Europe‐2003. Last Accessed 13th June 2023.

[8] Fitzmaurice C , Abate D , Abbasi N , Abbastabar H , Abd‐Allah F , Abdel‐Rahman O , et al. Global, regional, and national cancer incidence, mortality, years of life lost, years lived with disability, and disability‐adjusted life‐years for 29 cancer groups, 1990 to 2017: a systematic analysis for the global burden of disease study. JAMA Oncol. 2019;5(12):1749–1768. 10.1001/jamaoncol.2019.2996 31560378 PMC6777271

[9] Zhang W , Zeng W , Jiang A , He Z , Shen X , Dong X , et al. Global, regional and national incidence, mortality and disability‐adjusted life‐years of skin cancers and trend analysis from 1990 to 2019: an analysis of the global burden of disease study 2019. Cancer Med. 2021;10(14):4905–4922. 10.1002/cam4.4046 34105887 PMC8290243

[10] Guo A , Liu X , Li H , Cheng W , Song Y . The global, regional, national burden of cutaneous squamous cell carcinoma (1990–2019) and predictions to 2035. Eur J Cancer Care. 2023;2023:5484597–5484598. 10.1155/2023/5484597

[11] Hollestein LM , de Vries E , Aarts MJ , Schroten C , Nijsten TE . Burden of disease caused by keratinocyte cancer has increased in The Netherlands since 1989. J Am Acad Dermatol. 2014;71(5):896–903. 10.1016/j.jaad.2014.07.003 25190484

[12] World Health Organisization . Archived: WHO timeline ‐ COVID‐19. https://www.who.int/news/item/27‐04‐2020‐who‐timeline‐‐‐covid‐19. Last Accessed 7th August 2023.

[13] Baumann BC , MacArthur KM , Brewer JD , Mendenhall WM , Barker CA , Etzkorn JR , et al. Management of primary skin cancer during a pandemic: multidisciplinary recommendations. Cancer. 2020;126(17):3900–3906. 10.1002/cncr.32969 32478867 PMC7301000

[14] Di Gessa G , Price D . The impact of shielding during the COVID‐19 pandemic on mental health: evidence from the English Longitudinal Study of Ageing. Br J Psychiatry. 2022;221(4):637–643. 10.1192/bjp.2022.44 35369895 PMC11920597

[15] Sykes A , Pandit M . Experiences, challenges and lessons learnt in medical staff redeployment during response to COVID‐19. BMJ Lead. 2021;5(2):98–101. 10.1136/leader-2020-000313 37579288

[16] Angelini M , Teglia F , Astolfi L , Casolari G , Boffetta P . Decrease of cancer diagnosis during COVID‐19 pandemic: a systematic review and meta‐analysis. Eur J Epidemiol. 2023;38(1):31–38. 10.1007/s10654-022-00946-6 36593334 PMC9807424

[17] Tejera‐Vaquerizo A , Paradela S , Toll A , Santos‐Juanes J , Jaka A , López A , et al. Effects of COVID‐19 lockdown on tumour burden of melanoma and cutaneous squamous cell carcinoma. Acta Derm Venereol. 2021;101(8):adv00525. 10.2340/00015555-3890 34396424 PMC9413778

[18] Asai Y , Nguyen P , Hanna TP . Impact of the COVID‐19 pandemic on skin cancer diagnosis: a population‐based study. Public Library of Science. 2021;16(3):e0248492. 10.1371/journal.pone.0248492 PMC8011724 33788858

[19] Marson JW , Maner BS , Harding TP , Meisenheimer J, VII , Solomon JA , Leavitt M , et al. The magnitude of COVID‐19's effect on the timely management of melanoma and nonmelanoma skin cancers. J Am Acad Dermatol. 2021;84(4):1100–1103. 10.1016/j.jaad.2020.12.065 33482258 PMC7817517

[20] Lallas A , Kyrgidis A , Manoli S.‐M , Papageorgiou C , Lallas K , Sotiriou E , et al. Delayed skin cancer diagnosis in 2020 because of the COVID‐19‐related restrictions: data from an institutional registry. J Am Acad Dermatol. 2021;85:721–723. 10.1016/j.jaad.2021.05.021 34052332 PMC8156834

[21] Danesh MJ , Porter M , Brag K , Salian P , Olbricht S . COVID‐19 impacts on dermatologic surgery patients: a single institution experience. J Am Acad Dermatol. 2021;84(6):1698–1699. 10.1016/j.jaad.2021.02.060 33640504 PMC7906857

[22] Ferrara G , De Vincentiis L , Ambrosini‐Spaltro A , Barbareschi M , Bertolini V , Contato E , et al. Cancer diagnostic delay in northern and Central Italy during the 2020 lockdown due to the coronavirus disease 2019 pandemic. Am J Clin Pathol. 2021;155(1):64–68. 10.1093/ajcp/aqaa177 32995855 PMC7543252

[23] McClean A , Matteucci P , Totty J . The impact of COVID19 on the presentation, diagnosis and management of cutaneous melanoma and squamous cell carcinoma in a single tertiary referral centre. J Plast Reconstr Aesthetic Surg. 2022;75(8):2831–2870. 10.1016/j.bjps.2022.06.062 PMC9217629 35817712

[24] Ribes J , Pareja L , Sanz X , Mosteiro S , Escribà J , Esteban L , et al. Cancer diagnosis in Catalonia (Spain) after two years of COVID‐19 pandemic: an incomplete recovery. Eur Soc Med Oncol Open. 2022;7(3):100486. 10.1016/j.esmoop.2022.100486 PMC9197337 35714476

[25] Sangers TE , Wakkee M , Kramer‐Noels EC , Nijsten T , Louwman MW , Jaspars EH , et al. Limited impact of COVID‐19‐related diagnostic delay on cutaneous melanoma and squamous cell carcinoma tumour characteristics: a nationwide pathology registry analysis. Br J Dermatol. 2022;187(2):196–202. 10.1111/bjd.21050 35141890 PMC9111693

[26] Glasbey J , Ademuyiwa A , Adisa A , AlAmeer E , Arnaud AP , Ayasra F , et al. Effect of COVID‐19 pandemic lockdowns on planned cancer surgery for 15 tumour types in 61 countries: an international, prospective, cohort study. Lancet Oncol. 2021;22(11):1507–1517. 10.1016/s1470-2045(21)00493-9 34624250 PMC8492020

[27] Ahmed S , Sanghvi K , Yeo D . Telemedicine takes centre stage during COVID‐19 pandemic. BMJ Innovations. 2020;6:252–254. 10.1136/bmjinnov-2020-000440 37556278

[28] Rich H , Jones B , Malin I , Hemington‐Gorse SJ , Cubitt JJ . Plastic surgical management of skin cancer patients during the COVID‐19 pandemic. J Plast Reconstr Aesthetic Surg. 2021;74(3):644–710. 10.1016/j.bjps.2020.08.143 PMC7502181 33060056

[29] Roseleur J , Gonzalez‐Chica D , Emery J , Stocks N . Skin checks and skin cancer diagnosis in Australian general practice before and during the COVID‐19 pandemic, 2011‐2020. Br J Dermatol. 2021;185(4):853–855. 10.1111/bjd.20494 34009666 PMC8239661

[30] Ruggiero A , Martora F , Fabbrocini G , Villani A , Marasca C , Megna M , et al. The role of teledermatology during the COVID‐19 pandemic: a narrative review. Clin Cosmet Invest Dermatol. 2022;15:2785–2793. 10.2147/ccid.s377029 PMC9783831 36569420

[31] World Health Organization: IARC . Non‐melanoma skin cancer. https://gco.iarc.fr/today/data/factsheets/cancers/17‐Non‐melanoma‐skin‐cancer‐fact‐sheet.pdf. Last Accessed 8th July 2023.

[32] World Health Organization . WHO Director‐General's opening remarks at the media briefing on COVID‐19 ‐ 11 March 2020. https://www.who.int/director‐general/speeches/detail/who‐director‐general‐s‐opening‐remarks‐at‐the‐media‐briefing‐on‐covid‐19‐‐‐11‐march‐2020. Last Accessed 7th July 2023.

[33] Lefebvre C , Glanville J , Briscoe S . Chapter 4: searching for and selecting studies. https://training.cochrane.org/handbook. Last Accessed 15th February 2023.

[34] Yang D , Borsky K , Jani C , Crowley C , Rodrigues JN , Matin RN , et al. Trends in keratinocyte skin cancer incidence, mortality and burden of disease in 33 countries between 1990 and 2017. Br J Dermatol. 2022;188(2):237–246. 10.1093/bjd/ljac064 36763862

[35] Trakatelli M , Ulrich C , Del Marmol V , Euvrard S , Stockfleth E , Abeni D . Epidemiology of nonmelanoma skin cancer (NMSC) in Europe: accurate and comparable data are needed for effective public health monitoring and interventions. Br J Dermatol. 2007;156(s3):1–7. 10.1111/j.1365-2133.2007.07861.x 17488399

[36] Deeks J , Higgins J , Altman D . Chapter 10: analysing data and undertaking meta‐analyses. www.training.cochrane.org/handbook. Last Accessed 15th February 2023.

[37] The Cochrane Collaboration . Review Manager (RevMan). https://training.cochrane.org/online‐learning/core‐software/revman Last Accessed 7th July 2023.

[38] Higgins J , Li T , Deeks J . Chapter 6: choosing effect measures and computing estimates of effect. http://www.training.cochrane.org/handbook. Last Accessed 15th February 2023.

[39] Page M , McKenzie J , Bossuyt P , Boutron I , Hoffmann TC , Mulrow CD , et al. The PRISMA 2020 statement: an updated guideline for reporting systematic reviews. BMJ. 2021;372:71. 10.1136/bmj.n71 PMC8005924 33782057

[40] Ibrahim LS , Venables ZC , McPhail S , Levell NJ . Missing melanomas in England during the COVID‐19 pandemic: 2485 fewer melanoma diagnoses in 2020 than in 2019. Br J Dermatol. 2023;189(3):345–347. 10.1093/bjd/ljad117 37032445

[41] Vella C , Parvez W , Ashraf A , Ajmal S , Sudhir R , Agrawal S , et al. Changes in lung cancer staging and emergency presentations during the first year of the COVID‐19 pandemic. Chron Respir Dis. 2023;20:14799731231157770. 10.1177/14799731231157770 37564035 PMC10422907

[42] Green AC , Olsen CM . Cutaneous squamous cell carcinoma: an epidemiological review. Br J Dermatol. 2017;177(2):373–381. 10.1111/bjd.15324 28211039

[43] Mularczyk‐Tomczewska P , Zarnowski A , Gujski M , Jankowski M , Bojar I , Wdowiak A , et al. Barriers to accessing health services during the COVID‐19 pandemic in Poland: a nationwide cross‐sectional survey among 109,928 adults in Poland. Front Publ Health. 2022;10:986996. 10.3389/fpubh.2022.986996 PMC9495711 36159267

[44] Schachtel MJC , Gandhi M , Bowman JJ , Porceddu SV , Panizza BJ . Epidemiology and treatment outcomes of cutaneous squamous cell carcinoma extending to the temporal bone. Head Neck. 2022;44(12):2727–2743. 10.1002/hed.27185 36082824 PMC9826480

[45] Samarasinghe V , Madan V . Nonmelanoma skin cancer. J Cutan Aesthetic Surg. 2012;5(1):3–10. 10.4103/0974-2077.94323 PMC3339125 22557848

[46] Venables ZC , Ahmed S , O Bleiker T , Broggio J , Kwiatkowska M , Levell NJ , et al. The impact of the COVID‐19 pandemic on skin cancer incidence and treatment in England, 2020. Br J Dermatol. 2021;185:460–462. 10.1111/bjd.20409 33937975 PMC8239907

[47] Slotman E , Schreuder K , Nijsten TEC , Wakkee M , Hollestein L , Mooyaart A , et al. The impact of the COVID‐19 pandemic on keratinocyte carcinoma in The Netherlands: trends in diagnoses and magnitude of diagnostic delays. J Eur Acad Dermatol Venereol. 2022;36(5):680–687. 10.1111/jdv.17976 35092107

[48] National Institute for Health and Care Excellence (NICE) . Scenario: referral for suspected skin cancer. https://cks.nice.org.uk/topics/skin‐cancers‐recognition‐referral/management/referral‐for‐suspected‐skin‐cancer/#referral‐for‐suspected‐basal‐cell‐carcinoma. Last Accessed 9th August 2023.

[49] Watt T , Sullivan R , Aggarwal A . Primary care and cancer: an analysis of the impact and inequalities of the COVID‐19 pandemic on patient pathways. BMJ Open. 2022;12(3):e059374. 10.1136/bmjopen-2021-059374 PMC8948073 35332047

[50] Vila‐Payeras A , Domínguez C , Solà A , Nadal C , Taberner R . Incidental skin cancer detection in a hospital department: a prospective study. Actas Dermosifiliogr (English Edition). 2020;111(6):496–502. 10.1016/j.adengl.2020.04.013 32401722

[51] Omara S , Wen D , Ng B , Anand R , Matin RN , Taghipour K , et al. Identification of incidental skin cancers among adults referred to Dermatologists for suspicious skin lesions. JAMA Netw Open. 2020;3(12):e2030107–e. 10.1001/jamanetworkopen.2020.30107 33326027 PMC7745102

[52] World Health Organisization . The true death toll of COVID‐19: estimating global excess mortality. https://www.who.int/data/stories/the‐true‐death‐toll‐of‐covid‐19‐estimating‐global‐excess‐mortality. Last Accessed 3rd July 2023.

[53] World Health Organization . Global excess deaths associated with COVID‐19, January 2020 ‐ December 2021. https://www.who.int/data/stories/global‐excess‐deaths‐associated‐with‐covid‐19‐january‐2020‐december‐2021. Last Accessed 3rd July 2023.

[54] Ruggiero A , Martora F , Fornaro L , Guerrasio G , di Vico F , Fabbrocini G , et al. The impact of COVID‐19 pandemic on nonmelanoma skin cancers: report of a Southern Italy referral centre. Clin Exp Dermatol. 2022;47(11):2024–2025. 10.1111/ced.15307 35727878 PMC9349779

[55] Ruggiero A , Megna M , Fabbrocini G , Martora F . Video and telephone teledermatology consultations during COVID‐19 in comparison: patient satisfaction, doubts and concerns. Clin Exp Dermatol. 2022;47(10):1863–1864. 10.1111/ced.15286 35656801 PMC9347843

[56] NHS England . NHS expands high‐res skin imaging to speed up cancer diagnoses and treatment for tens of thousands of patients. https://www.england.nhs.uk/2023/06/nhs‐expands‐high‐res‐skin‐imaging‐to‐speed‐up‐cancer‐diagnoses‐and‐treatment‐for‐tens‐of‐thousands‐of‐patients/. Last Accessed 4th July 2023.

[57] Shahid S , Gao J , Corriero AC , Roszpopa J , Miranda BH . A study of the effects of delayed patient presentation on cutaneous SCC progression. J Plast Reconstr Aesthetic Surg. 2022;75(2):722–729. 10.1016/j.bjps.2021.09.015 34844882

[58] Balakirski G , Sabulyte S , Wesselmann U , Michalowitz A , Kreuter A , Hofmann SC . Long‐term effects of the COVID‐19 pandemic on cutaneous squamous cell carcinoma: increase of thick tumors in two German dermatology clinics. JDDG J der Deutschen Dermatol Gesellschaft. 2023;21(8):910–913. 10.1111/ddg.15087 37186057

[59] Jović M , Marinković M , Suđecki B , Jurišić M , Bukumirić Z , Jovanović M , et al. COVID‐19 and cutaneous squamous cell carcinoma‐impact of the pandemic on unequal access to healthcare. Healthcare. 2023;11(14):1994. 10.3390/healthcare11141994 37510435 PMC10378852

[60] Yanez ND , Weiss NS , Romand J.‐A , Treggiari MM . COVID‐19 mortality risk for older men and women. BMC Publ Health. 2020;20(1):1742. 10.1186/s12889-020-09826-8 PMC7675386 33213391

[61] Sinikumpu SP , Jokelainen J , Keinänen‐Kiukaanniemi S , Huilaja L . Skin cancers and their risk factors in older persons: a population‐based study. BMC Geriatr. 2022;22(1):269. 10.1186/s12877-022-02964-1 35361154 PMC8973875

[62] Drescher CW , Bograd AJ , Chang S.‐C , Weerasinghe RK , Vita A , Bell RB . Cancer case trends following the onset of the COVID‐19 pandemic: a community‐based observational study with extended follow‐up. Cancer. 2022;128(7):1475–1482. 10.1002/cncr.34067 34919267

[63] Soerjomataram I , Bardot A , Aitken J , Piñeros M , Znaor A , Steliarova‐Foucher E , et al. Impact of the COVID‐19 pandemic on population‐based cancer registry. Int J Cancer. 2022;150(2):273–278. 10.1002/ijc.33792 34480348 PMC8652711

[64] Iacobucci G . Pandemic has accelerated demand for private healthcare, report finds. BMJ. 2022;376:o566. 10.1136/bmj.o566 35241427

[65] Boutron I , Page MJ , Higgins JPT , et al. Chapter 7: considering bias and conflicts of interest among the included studies. http://www.training.cochrane.org/handbook. Last Accessed 8th May 2024.

[66] GOV.UK . National cancer registration and analysis service (NCRAS). https://www.gov.uk/guidance/national‐cancer‐registration‐and‐analysis‐service‐ncras. Last Accessed 9th August 2023.

[67] GOV.UK . Staying at home and away from others (social distancing). https://www.gov.uk/government/publications/full‐guidance‐on‐staying‐at‐home‐and‐away‐from‐others/full‐guidance‐on‐staying‐at‐home‐and‐away‐from‐others. Last Accessed 9th August 2023.

[68] Seité S , Del Marmol V , Moyal D , Friedman A . Public primary and secondary skin cancer prevention, perceptions and knowledge: an international cross‐sectional survey. J Eur Acad Dermatol Venereol. 2017;31(5):815–820. 10.1111/jdv.14104 28045207 PMC6084324

[69] James Lind Alliance . Skin cancer surgery top 10. https://www.jla.nihr.ac.uk/priority‐setting‐partnerships/skin‐cancer‐surgery/top‐10‐priorities.htm. Last Accessed 8th August 2023.

[70] Menzies S , Forristal H , Hennessy C , Yeates L , Ormond P . Public awareness of skin cancer: results of a large national survey in Ireland. Ir J Med Sci. 2017;186(1):73–74. 10.1007/s11845-016-1481-z 27423651

[71] Heerfordt IM . Decreased public awareness of skin cancer during the coronavirus pandemic. Int J Dermatol. 2021;60(9):e334. 10.1111/ijd.15659 33982801 PMC8239598

[72] Miranda BH , Jica RCI , Pinto‐Lopes R , Mopuri N , Sood MK , Tare M , et al. St andrew’s COVID‐19 surgery safety (StACS) study: skin cancer. J. Plast. Surg. Hand Surg. 2021;55(5):315–321. 10.1080/2000656x.2021.1883633 33606568

