# Supplementary material for: Impact of the COVID‐19 pandemic on international cutaneous squamous cell carcinoma incidence: A systematic review and meta‐analysis
Source: Skin Health Dis. 2024 Jun 15;4(4):e405. doi: 10.1002/ski2.405 (PMC11297431; doi:10.1002/ski2.405)
Supplement: Supplementary file 1 — Supporting Information S1 [file SKI2-4-e405-s001.docx]

**Table S1:** search strategy including four bibliographic databases and grey literature

| **EBSCO MEDLINE** | 1. (MH”skin Neoplasms”) 2. “squamous cell carcinoma*”- (“Tx All text” applied to all below) 3. “cutaneous squamous cell carcinoma*” 4. “Non melanoma skin cancer*” 5. “Keratinocyte cancer*” 6. NMSC 7. SCC 8. cSCC 9. “Skin neoplasm*” 10. “Skin carcinoma*” 11. “Skin cancer*” 12. OR/1-11 13. “Epidemiology or incidence or prevalence or occurrence”- 14. “Incidence rate*” 15. 13 OR 14 16. COVID* 17. Covid-19 or coronavirus or 2019-ncov or sars-cov2 or cov-19 18. 16 OR 17 19. 12 AND 18 20. 12 AND 15 AND 18 with 2020-2023 date restriction applied |
| --- | --- |
| **EBSCO CINAHL** | 1. (MH”Skin neoplasms”)- (subject heading) 2. “Skin neoplasm*” (“Tx All text” applied to all below) 3. “Skin cancer*” 4. “Skin carcinoma*” 5. “Keratinocyte cancer*” 6. “Non melanoma skin cancer*” 7. “Cutaneous squamous cell carcinoma*” 8. “Squamous cell carcinoma*” 9. NMSC 10. SCC 11. cSCC 12. OR/1-11 13. COVID* (Tx All text applied to all) 14. Covid-19 or coronavirus or 2019-ncov or sars-cov2 or cov-19 15. 13 OR 14 16. “Epidemiology or incidence or prevalence or occurrence”- TX All text for all 17. “Incidence rate*” 18. 16 OR 17 19. 12 AND 15 AND 18 with 2020-2023 data restriction range applied |
| **OVID Embase- basic search** | 1. exp skin tumor/ 2. Exp skin carcinoma/ 3. “Non-melanoma skin cancer*” 4. NMSC 5. “Cutaneous squamous cell carcinoma*” 6. cSCC 7. “Skin neoplasm*” 8. “Keratinocyte cancer*” 9. “Squamous cell carcinoma*” 10. SCC 11. OR/1-10 12. “Incidence rate*” 13. Epidemiology 14. prognosis 15. OR/12-14 16. COVID* - (only one with advanced search) 17. SARS-COV-2 18. 16 OR 17 19. 11 AND 15 20. 11 AND 18 21. 19 OR 20 with 2020-2023 date restriction applied |
| **Webofscience** | 1. ALL= (“Skin cancer*”) 2. ALL= (“Skin neoplasm*”) 3. ALL= “Skin carcinoma*” 4. ALL= (“Squamous cell carcinoma*”) 5. ALL= (“Cutaneous squamous cell carcinoma*”) 6. ALL= ("non melanoma skin cancer*") 7. ALL= (“Keratinocyte cancer*”) 8. ALL= (SCC) 9. ALL= (cSCC) 10. ALL= (NMSC) 11. OR/1-10 ( 12. ALL= (“COVID*”) 13. ALL= (“coronavirus*”) 14. 12 OR 13 15. ALL= (“incidence*”) 16. ALL= (“prevalence*”) 17. ALL= (“Epidemiology*”) 18. OR/15-17 19. 11 AND 14 AND 18 with 2020-2023 date restriction applied |
| **Google scholar** | Two separate searches were carried out on Google Scholar, URL: <https://scholar.google.com>. The first 100 results were screened, sorted by relevance, using the following key phrases:   1. “Cutaneous squamous carcinoma cell incidence COVID-19”. 2. “Non melanoma skin cancer incidence COVID-19” |
| **National cancer registries searched on Google.** | 33 separate searches were carried out on Google, URL: <https://www.google.com>. The first 5 results of each search were screened for relevant government reports or cancer registry websites using the key phrase:  “National cancer registry X”  X= represents a different country. The 33 countries searched which were classified as having high-quality epidemiological data^34^ were: Australia, Austria, Belgium, Bulgaria, Canada, Croatia, Czech Republic, Denmark, Estonia, Finland, France, Germany, Greece, Hungary, Iceland, Ireland, Italy, Japan, Latvia, Lithuania, Luxembourg, Malta, Netherlands, Norway, Poland, Portugal, Romania, Slovenia, Spain, Sweden, Switzerland, United Kingdom, United States. |

**Table S2:** Summary of A.Lomas quality assessment tool results using with corresponding questions provided below

| **Author, Year** | **Q1** | **Q2** | **Q3** | **Q4** | **Q5** | **Q6** | **Q7** | **Q8** | **Q9** | **Q10** | **Total/10** |
| --- | --- | --- | --- | --- | --- | --- | --- | --- | --- | --- | --- |
| Belgium, 2023 | 0 | 1 | 1 | 1 | 1 | 1 | 1 | 1 | 0 | 1 | 8 |
| Biscgelia, 2022 | 0 | 1 | 1 | 0 | 0 | 0 | 0 | 0 | 0 | 0 | 2 |
| Denmark, 2023 | 0 | 1 | 1 | 0.5 | 0 | 1 | 0 | 0 | 0 | 0 | 3.5 |
| NHS Digital, 2022 | 0 | 1 | 1 | 0 | 0 | 1 | 1 | 1 | 0 | 1 | 6 |
| NCR, 2022 | 0 | 1 | 1 | 1 | 1 | 1 | 1 | 0 | 0 | 1 | 7 |
| NORDCAN, Iceland, 2022 | 0 | 1 | 1 | 0.5 | 1 | 1 | 1 | 0 | 0 | 1 | 6.5 |
| NORDCAN, Sweden, 2022 | 0 | 1 | 1 | 0.5 | 1 | 1 | 1 | 0 | 0 | 1 | 6.5 |
| Northern Ireland, 2020 | 0 | 1 | 1 | 0 | 1 | 1 | 1 | 1 | 0 | 1 | 7 |
| Norway, 2021 | 0 | 1 | 1 | 0.5 | 1 | 1 | 1 | 0 | 0 | 1 | 6.5 |
| Pitkäniemi J, 2020 | 0 | 1 | 1 | 1 | 1 | 1 | 1 | 0 | 0 | 0 | 6 |
| Ribes, 2022 | 0 | 1 | 1 | 1 | 1 | 1 | 0 | 1 | 0 | 0 | 6 |
| Saarland, 2022 | 0 | 1 | 1 | 0 | 1 | 1 | 1 | 0 | 0 | 1 | 6 |
| Schleswig-Holstein, 2022 | 0 | 1 | 1 | 1 | 1 | 1 | 1 | 0 | 0 | 1 | 7 |
| Scotland, 2021 | 0 | 1 | 1 | 1 | 1 | 1 | 1 | 1 | 0 | 1 | 8 |

**Internal validity:**

**Data collection:**

Q1: Did the study directly sample the population, as opposed to using cancer registries?

**Description of methods**:

Q2: Is the method of counting tumours stated?

Q3: Is there histological verification?

**Reporting of incidence rates:**

Q4: Were separate rates reported for BCC and SCC?

Q5: Are gender-specific incidence rates reported?

Q6: Are age-specific incidence rates reported?

Q7: Were the rates age standardised?

Q8: Are confidence intervals used?

**External validity:**

Q9: Is there information about the ethnicity or skin type of the population?

Q10: Were the data standardised to a major population

**Table S3:** cSCC and NMSC crude incidence rate ratios of all fourteen studies included

| **Author, Year** | **Country** | **Diagnosis** | **COVID-19  Time interval(s)** | **2019 Crude incidence rate  per 100,000 PY** | **Crude incidence rate(s) during COVID-19 per 100,000 PY** | **Crude Incidence rate ratio(s)** |
| --- | --- | --- | --- | --- | --- | --- |
| Belgium  2023 | Belgium | cSCC  and NMSC | 01/2020- 12/2020 | **cSCC** B= 75.4, M=90.8,  F= 60.5 **(a)** **NMSC** B= 368.92,  M= 375.4, F= 363.3 | **cSCC** B= 76.5,  M= 91.7, F= 61.8 **NMSC** B= 341.9, M= 349.2,  F= 335.4 | **cSCC** B= 1.01,  M= 1.01,  F= 1.02 **NMSC** B= 0.93, M= 0.93, F= 0.92 |
| Biscgelia,2022 | Italy | NMSC | 01/2020- 12/2020 | B= 212.97 **(b)** | B= 160.15 | B= 0.75 |
| Denmark,  2023 | Denmark | ICD-10 C44  exc. BCC | **1)** 01/2020- 12/2020 **2)** 01/2021- 12/2021 | B= 63.74 | **1)** B= 80.19 **2)** B= 86.53 | **1)** B= 1.26 **2)** B= 1.36 |
| NHS Digital 2022 | England | NMSC | 01/2020- 12/2020 | B= 232.1, M= 265.1 F= 199.9 | B= 184.5, M= 209.4 F= 157.9 | B= 0.79, M= 0.79 F= 0.79 |
| NCR 2022 | Netherlands | cSCC | **1)** 01/2020- 12/2020 **2)** 01/2021- 12/2021 **3)** 01/2022- 12/2022 | B= 81.59 M= 90.29 F= 73.01 | **1)** B= 80.30,  M= 88.70, F=72.01 **2)** B= 84.24, M= 93.73, F= 74.86 **3)** B= 84.55, M= 94.39, F= 74.82 | **1)** B= 0.98,  M= 0.98, F= 0.99 **2)** B= 1.03,  M= 1.04, F= 1.03 **3)** B= 1.04,  M= 1.05, F= 1.02 |
| NORDCAN 2022 | Iceland | ICD-10 C44  exc. BCC | 01/2020- 12/2020 | B= 42.58, M= 47.1, F= 37.0 | B= 40.37, M= 40.9,  F= 39.4 | B= 0.95, M= 0.87, F= 1.07 |
| NORDCAN 2022 | Sweden | ICD-10 C44  exc. BCC | 01/2020- 12/2020 | B= 59.92, M=65.9, F=53.9 | B= 61.45, M= 66.6, F= 56.3 | B= 1.03, M= 1.01, F= 1.04 |
| Northern Ireland 2020 | Northern  Ireland | NMSC | 01/2020- 12/2020 | B= 226.5, M= 264.0, F= 190.1 | B= 167.3, M= 201.8, F= 133.9 | B= 0.74, M= 0.76, F= 0.70 |
| Norway 2021 | Norway | ICD-10 C44  exc. BCC | **1)** 01/2020- 12/2020 **2)** 01/2021- 12/2021 **3)** 01/2022- 12/2022 | B= 49.20, M= 52.9, F= 45.5 | **1)** B= 52.57, M=56.0, F= 49.1 **2)** B= 53.95, M=58.1, F= 49.8, **3)** B= 56.10, M= 61.3, F= 50.8 | **1)** B= 1.07, M=1.06,  F= 1.08 **2)** B= 1.10, M= 1.10, F= 1.09 **3)** B= 1.14, M= 1.16, F= 1.11 |
| Pitkäniemi  2020 | Finland | cSCC | 01/2020- 12/2020 | B= 34.3, M= 38.9 F= 29.8 | B= 34.7, M= 37.8 F= 31.6 | B= 1.01, M= 0.97 F= 1.06 |
| Ribes 2022 | Spain | cSCC | **1)** 01/2020- 12/2020 **2)** 01/2021- 12/2021 | **1)** B= 37.34, M=45.25, F= 29.74 | **1)** B= 31.11, M= 37.57, F= 24.88 **2)** B= 36.63, M= 42.08, F= 31.37 | **1)** B= 0.83, M= 0.83, F= 0.84 **2)** B= 0.98, M= 0.93, F= 1.05 |
| Saarland  2022 | Germany | NMSC | 01/2020- 12/2020 | B= 262.41, M= 284.2 F= 241.4 | B= 239.26, M= 260.9 F= 218.4 | B= 0.91, M= 0.92 F= 0.90 |
| Schleswig-Holstein 2022 | Germany | cSCC and NMSC | **1)** 01/2020- 12/2020 **2)** 01/2021- 12/2021 | **cSCC**  B= 77.20, M= 92.53, F= 62.47  **NMSC**  B= 290.34, M= 306.3 F= 275.0 | **cSCC**  **1)** B= 70.06, M= 84.25,  F= 56.44  **2)** B= 71.63, M= 84.78,  F= 59.00  **NMSC**  **1)** B= 258.71, M= 278.6, F= 239.6 **2)** B= 259.66, M= 274.2, F= 245.7 | **cSCC**  **1)** B= 0.91, M= 0.91,  F= 0.90  **2)** B= 0.93, M= 0.92,  F= 0.94  **NMSC**  **1)** B= 0.89, M= 0.91, F= 0.87 **2)** B= 0.89, M= 0.90, F= 0.89 |
| Scotland  2021 | Scotland | cSCC  and NMSC | **1)** 01/2020- 12/2020 **2)** 01/2021- 12/2021 | **cSCC** **1)** B= 68.58, M= 99.17, F= 39.50  **NMSC** B= 222.92, M= 269.28, F= 178.84 | **cSCC** **1)** B= 66.59, M= 95.94, F= 38.68 **2)** B= 76.94, M= 111.95, F= 43.6 **NMSC** **1)** B= 174.48, M= 216.68 F= 134.32 **2)** B= 204.22, M= 253.80 F= 157.02 | **cSCC** **1)** B= 0.97, M= 0.97, F= 0.98 **2)** B= 1.12, M= 1.13, F= 1.10 **NMSC** **1)** B= 0.78, M= 0.80 F= 0.75 **2)** B= 0.92, M= 0.94, F= 0.88 |

(a)- Genders: B, both male and female data recorded together; M, male only; F, female only

(b)- Studies states population area covered 532,000 which is applied to 2020 and 2019 NMSC count

**Table S4:** Age-standardised cSCC and NMSC incidence rate ratios of all eleven studies included in analysis

| **Author, Year** | **Country** | **Diagnosis** | **COVID-19  Time interval(s)** | **Age-standardisation  methods (a)** | **2019 Age-standardised incidence rate per 100,000 PY** | **Age-standardised incidence rate(s) during COVID-19 per 100,000 PY** | **Age-standardised incidence  rate RR ratio(s)** |
| --- | --- | --- | --- | --- | --- | --- | --- |
| Belgium, 2023 | Belgium | cSCC and NMSC | 01/2020- 12/2020 | **ESP (2013) reported** WSP | **cSCC**  B= 74.5, M= 108.4, F= 52.6 (b)  **NMSC**  B= 371.9, M= 425.2, F= 342.8 | **cSCC**  B= 74.9, M= 106.7, F= 53.8 **NMSC**  B= 341.9, M= 390.1, F= 315.1 | **cSCC**  B= 1.01, M= 0.98, F= 1.02  **NMSC**  B= 0.92, M= 0.92, F= 0.92 |
| NHS Digital, 2022 | England | NMSC | 01/2020- 12/2020 | ESP, 2013 | B= 244.5, M= 303.8, F=196.1 | B= 191.8, M= 238.0 F=153.8 | B= 0.78, M= 0.78, F= 0.78 |
| NCR, 2022 | Netherlands | cSCC | **1)** 01/2020-12/2020 **2)** 01/2021-12/2021 **3)** 01/2022-12/2022 | **ESP (2013) reported** WSP | B= 84.2, M= 106.32,  F= 68.3 | **1)** B= 81.67, M= 102.51, F= 66.65 **2)** B= 84.22, M= 105.55, F= 68.29 **3)** B= 83.96, M= 105.85, F= 67.7 | **1)** B= 0.97, M= 0.96, F= 0.97 **2)** B= 1.00, M= 0.99, F= 1.00 **3)** B= 1.00, M= 1.00, F= 0.99 |
| NORDCAN, 2022 | Iceland | ICD-10 C44  exc. BCC | 01/2020- 12/2020 | ESP, NORDCAN (2000) | M=37.4, F= 25.6 | M= 30.4, F= 26.6 | M= 0.81, F=1.04 |
| NORDCAN 2022 | Sweden | ICD-10 C44  exc. BCC | 01/2020- 12/2020 | ESP, NORDCAN | M= 38.4, F=26.6 | M= 38.1, F=27.7 | M= 0.99, F=1.04 |
| Northern Ireland, 2020 | Northern  Ireland | NMSC | 01/2020- 12/2020 | ESP, 2013 | B= 257.7, M= 333.0 F= 201.0 | B= 187.8, M= 250.6, F= 139.4 | B= 0.73, M= 0.75, F= 0.69 |
| Norway, 2021 | Norway | ICD-10 C44  exc. BCC | **1)** 01/2020-12/2020 **2)** 01/2021-12/2021 **3)** 01/2022-12/2022 | **ESP 1976 reported** WSP | M= 35.9, F= 25.1 | **1)** M= 36.7, F= 25.7 **2)** M= 37.0, F= 25.8 **3)** M= 38.5, F= 26.4 | **1)** M= 1.02, F=1.02 **2)** M= 1.03, F= 1.03 **3)** M= 1.07, F= 1.05 |
| Pitkäniemi,  2020 | Finland | cSCC | 01/2020- 12/2020 | **Finland (2014) reported** WSP | B= 30.7, M= 42.7 F= 22.8 | B= 30.6, M= 40.8, F= 24.3 | B= 1.00, M= 0.96, F= 1.07 |
| Saarland,2022 | Germany | NMSC | 01/2020- 12/2020 | ESP 1976 | M= 159.2, F= 128.3 | M= 143.4, F= 115.9 | M= 0.90, F= 0.90 |
| Schleswig-Holstein, 2022 | Germany | cSCC and NMSC | **1)** 01/2020-12/2020 **2)** 01/2021-12/2021 | ESP 1976 | **cSCC**  M= 44.90, F= 24.8  **NMSC**  M= 167.5, F= 143.7 | **cSCC**  1) M= 39.9, F= 22.4  2) M= 40.3, F= 23.9  **NMSC**  **1)** M= 152.4, F= 124.2 **2)** M= 150.0, F= 129.4 | **cSCC**  1) M= 0.89, F= 0.90  2) M= 0.90, F= 0.96  **NMSC**  **1)** M= 0.91, F= 0.86 **2)** M= 0.90, F= 0.90 |
| Scotland  2021 | Scotland | cSCC  and NMSC | **1)** 01/2020-12/2020 **2)** 01/2021-12/2021 | **ESP 2013 (reported)** WSP | **cSCC** B= 77.49, M= 118.87, F= 36.10 **NMSC** B= 236.65, M= 304.29,  F= 169.01 | **cSCC** **1)** B= 74.89, M= 114.57, F= 35.21 **2)** B= 84.80, M= 130.38, F= 39.21 **NMSC**: **1)** B= 185.06, M= 244.65, F=125.47  **2)** B= 213.67, M=281.84, F=145.49 | **cSCC** **1)** B= 0.97, M= 0.96, F= 0.97 **2)** B= 1.09, M= 1.10, F= 1.09 **NMSC**: **1)** B= 0.78, M= 0.80, F= 0.74 **2)** B= 0.90, M= 0.93, F= 0.86 |

(a)- Age-standardisation Rates: ESP, European standard population; WSP, World Standard population O, Other

(b)- Genders: B, both male and female data recorded together; M, male; F, Female
